# A comparative study of pleural effusion in water area, water temperature and postmortem interval in forensic autopsy cases of drowning

**DOI:** 10.1038/s41598-021-01047-2

**Published:** 2021-11-02

**Authors:** Akiko Ishigami, Masayuki Kashiwagi, Yuko Ishida, Kenji Hara, Mizuho Nosaka, Aya Matsusue, Hiroki Yamamoto, Brian Waters, Toshikazu Kondo, Shin-ichi Kubo

**Affiliations:** 1 Department of Forensic Medicine, Wakayama Medical University, 811-1 Kimiidera, Wakayama, 641-8509 Japan; 2 Department of Forensic Medicine, Fukuoka University, 7-45-1 Nanakuma, Fukuoka, 814-0180 Japan

**Keywords:** Respiration, Outcomes research

## Abstract

Japan is surrounded by the sea and is also a mountainous country with many rivers. Japan has the second- highest rate of deaths caused by drowning in the world. Pleural effusion (PE) is one of the major findings at autopsy. It is found in approximately 80% of drowning mortalities and is observable for a relatively long postmortem interval (PMI). We focused on the amount of pleural fluid in drowning cases, discussed the relationship of PE with the drowning environment, water temperature, and postmortem interval, and established more simple and practical criteria for the diagnosis of drowning. We measured the weight of the lungs, PE, and their sum as the intrathoracic (IT) weight (total weight of lungs + pleural effusion), and calculated the PE ratio [(PE weight/IT weight) × 100]. A total of 130 drowning deaths diagnosed through forensic autopsies were investigated in this study. The cases were classified by drowning environment (freshwater, brackish water, and seawater), water temperature (under 20 °C, more than 20 °C), and postmortem interval (less than 1 day, 1–3 days, more than 3 days). The present study demonstrated that the PE ratio may be more effective for the diagnosis of drowning. Moreover, the accumulation of PE is affected by drowning environment, water temperature, and PMI. Collectively, it is important to assess the PE ratio and consider these factors in autopsy cases of victims found in water.

## Introduction

Japan is surrounded by the sea and is also a mountainous country with many rivers. In Japan, the number of deaths caused by drowning is the second highest in the world^1^. External foam protruding from the nostrils and mouth, frothy fluid in the airways, lung overinflation and waterlogging, and pleural effusion (PE) are major findings in drowning^2,3^. However, these findings are not consistent. In particular, external foam cannot be observed in victims with a long postmortem interval (PMI). In contrast, PE is found in approximately 80% of drowning victims, and the observable time is relatively long^4^. Several lines of accumulating evidence suggested that the drowning index, which is the weight ratio of the lungs and PE to the spleen, is a useful indicator for the diagnosis of drowning^5,6^. However, spleen weight is often influenced by putrefaction as postmortem changes and/or antemortem disease conditions, such as splenomegaly^7^. Thus, forensic pathologists are required to explore other practical and reliable markers for the diagnosis of drowning. Moreover, cases of drowning in different types of environments, such as seawater, freshwater, and brackish water, show different pathophysiological features. In this study, we focused on the amount of pleural fluid in drowning cases, discussed the relationship of PE with the drowning environment, water temperature, and postmortem interval, and established more simple and practical criteria for the diagnosis of drowning.

## Result

### Drowning environment

**Figure 1:**
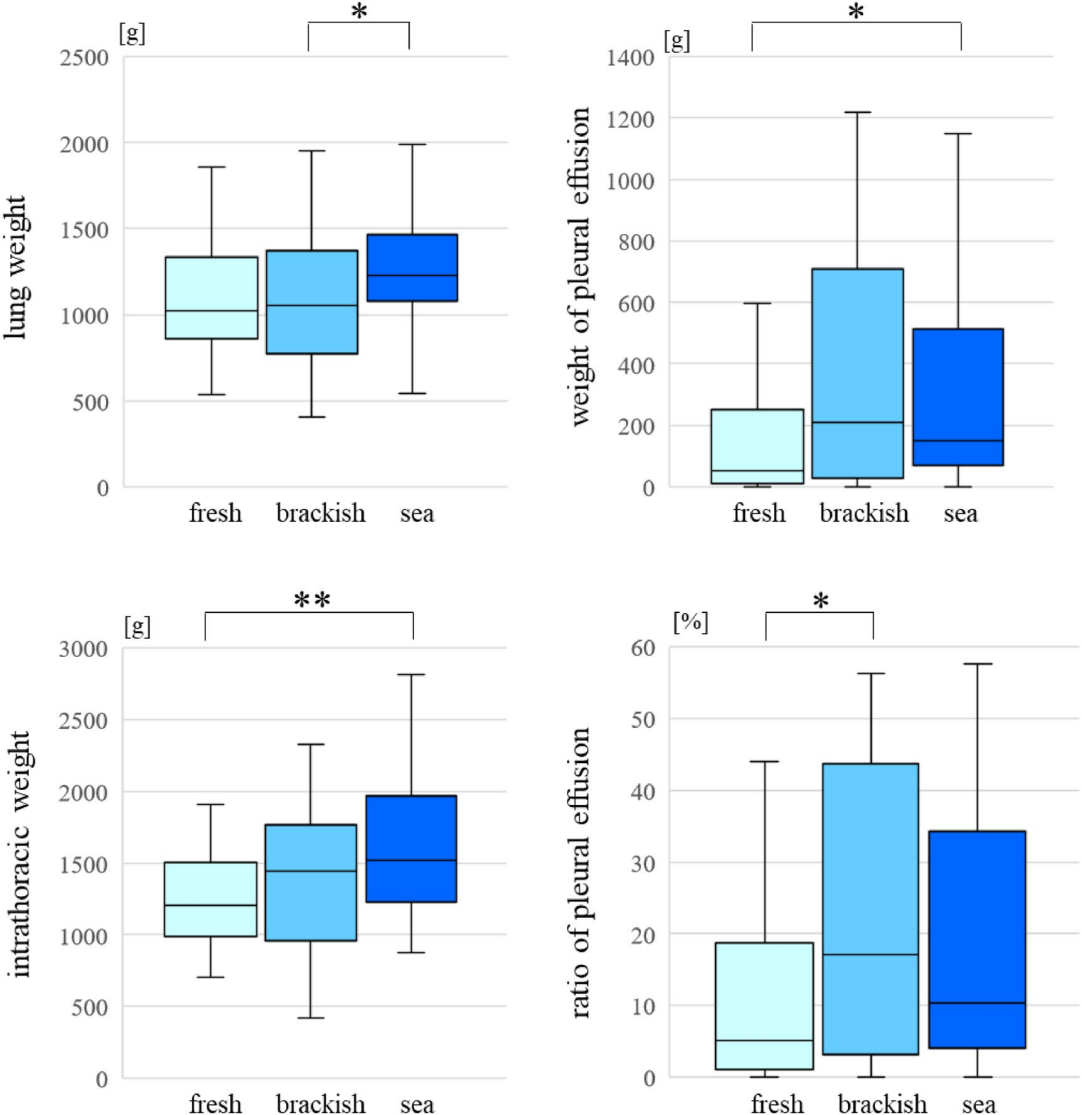
Comparing the parameters for different drowning environments.

The weight of the lungs was higher in the seawater group than in the brackish water group (p < 0.05). The weight of PE was higher in the seawater group than in the freshwater group (p < 0.05), as was the intrathoracic weight (p < 0.01). The PE ratio was lower in the freshwater group than in the brackish water group (p < 0.05) (Fig. 1). There were not any differences in each factor among the other groups.

### Postmortem interval (PMI)

**Figure 2:**
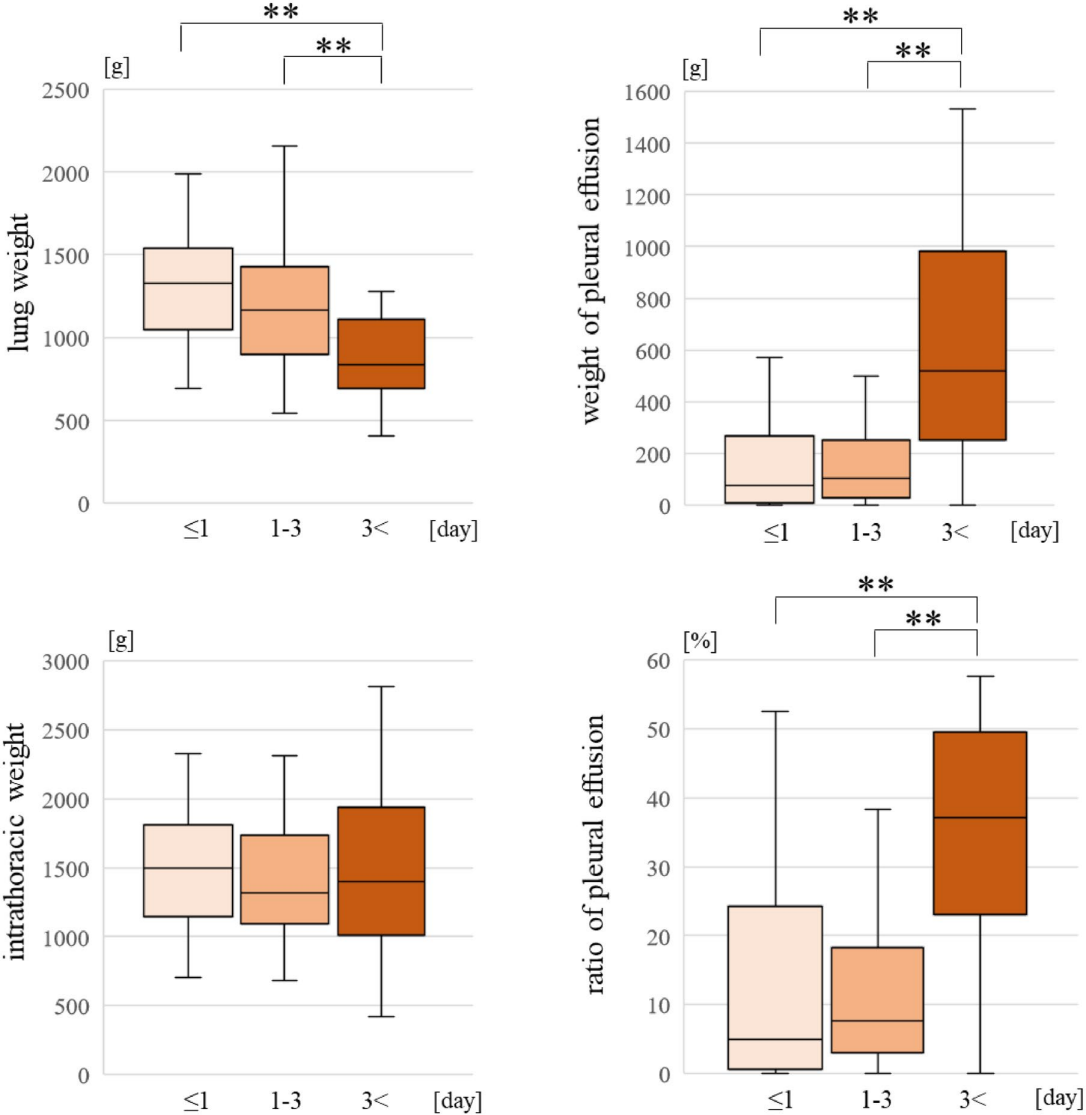
Comparing the parameter for different postmortem intervals.

The weight of the lungs, the weight of pleural fluid, and the PE ratio were highest in the ≥ 3 days group (p < 0.01) (Fig. 2).

### PMI and drowning environment (Table 1)

**Figure 3:**
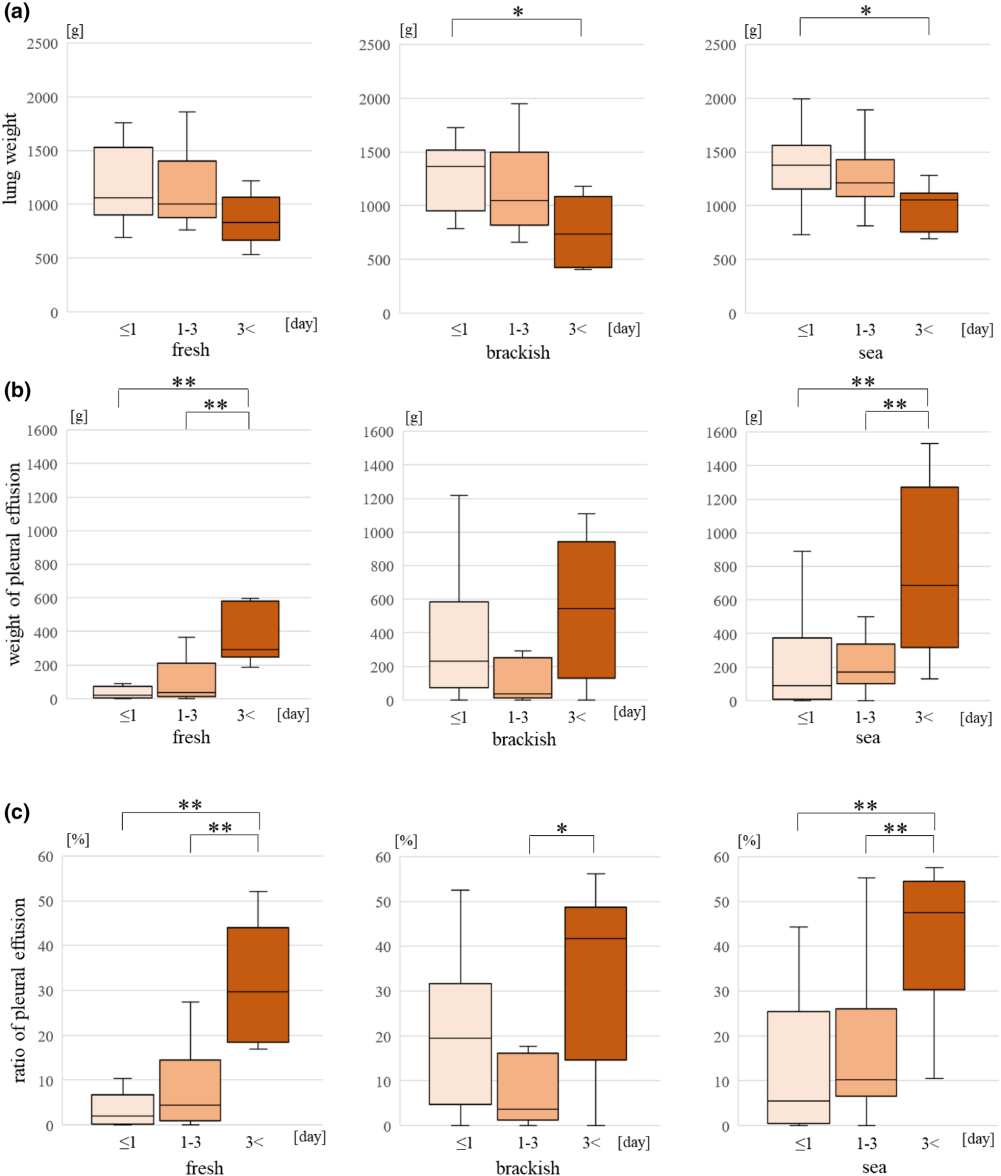
Comparing different parameters for both the drowning environment and postmortem interval **(a)** The weight of the lungs, **(b)** the weight of pleural effusion, **(c)** the ratio of pleural effusion.

1. Lung weight
  The weight of the lungs in the ≤ 1 day group was *heavier* than that in the ≥ 3 days group in brackish water and seawater (p < 0.05) (Fig. 3a).
2. PE weight
  The PE weight was higher in freshwater and seawater and the PMI was long (p < 0.01) (Fig. 3b).
3. PE ratio
  The PE ratio increased in freshwater and seawater as the PMI increased. In brackish water, it was significantly increased in the ≥ 3 days group than the 1–3 days group (Fig. 3c).

**Table 1:** The case number classified to PMI and drowning environment.

PMI	Within 1 day	From 1 to 3 days	Over 3 days
**Drowning environment**			
Fresh	13	14	7
Brackish	9	11	10
Sea	28	27	11

### Water temperature

**Figure 4:**
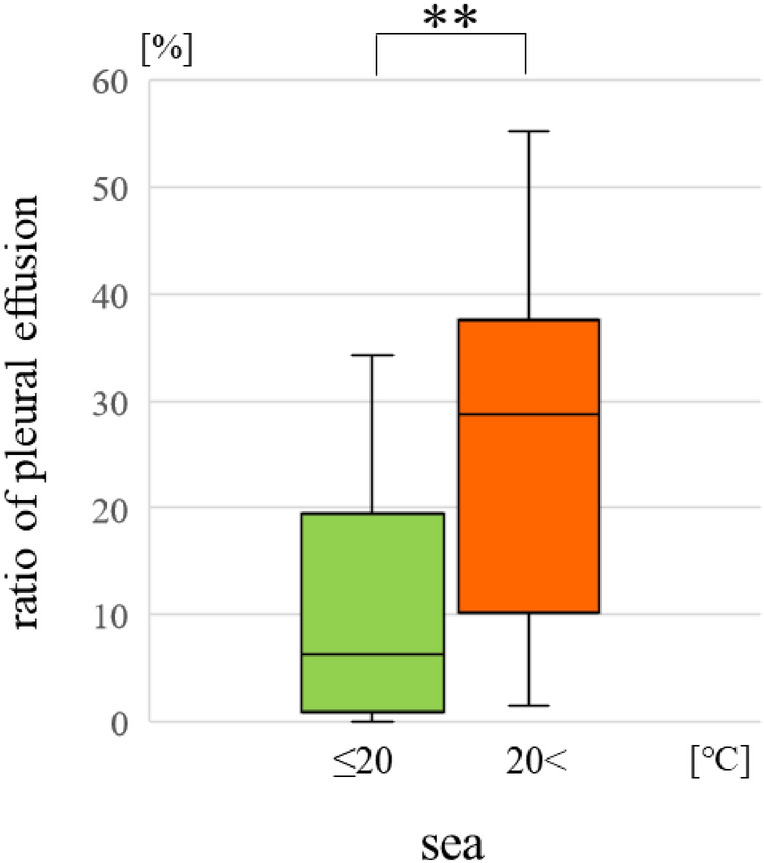
Comparing the intrathoracic weight and water temperature in the sea water group.

1. All cases classified by water temperature
  Each parameter showed no significant difference.
2. Drowning environment (Table 2)
  The PE ratio was higher in the < 20 °C group than in the ≤ 20 °C group in seawater (p < 0.01) (Fig. 4).

### PMI and water temperature (Table 3)

**Table 2:** The case number classified to water temperature and drowning environment.

Water temperature	Under 20 ℃	Over 20 ℃
**Drowning environment**		
Fresh	23	11
Brackish	21	9
Sea	40	26

**Table 3:** The case number classified to PMI and water temperature.

PMI	Within 1 day	From 1 to 3 days	over 3 days
**Water temperature**			
Under 20 ℃	32	35	17
Over 20 ℃	18	17	11

**Figure 5:**
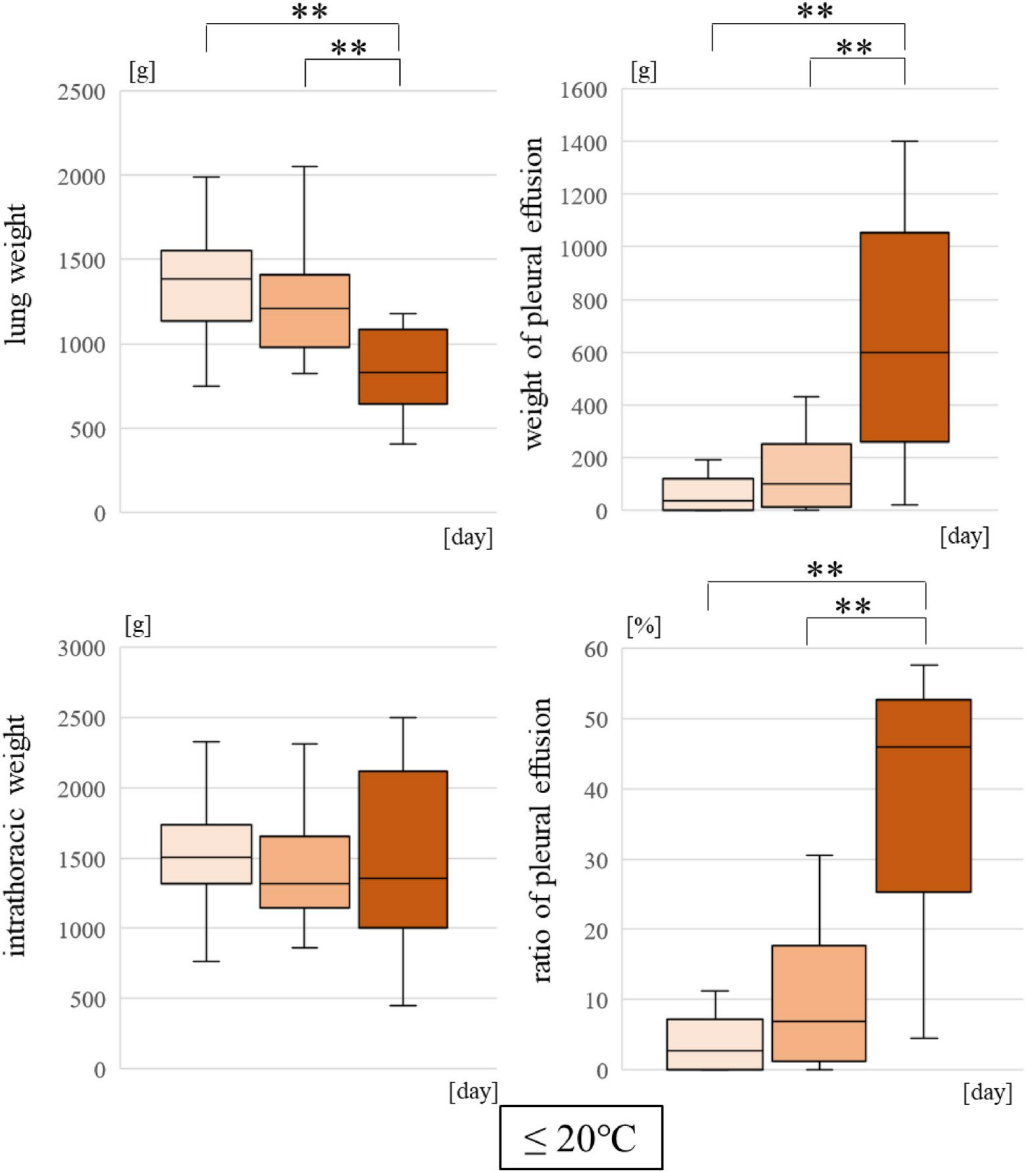
Comparing the parameters for different postmortem intervals in the lower water temperature group.

The weight of the lungs decreased and the weight of PE increased as the PMI increased. The PE ratio also increased. These changes were conspicuous in the ≤ 20 °C group (p < 0.01) (Fig. 5). In the 20 °C < group, there was no significant difference.

### Drowning environment, water temperature, and PMI (Table 4)

**Table 4:** The case number classified to drowning environment, water temperature, and PMI.

Drowning environment	Fresh		Brackish		Sea	
Temperature	Under 20 ℃	Over 20 ℃	Under 20 ℃	Over 20 ℃	Under 20 ℃	Over 20 ℃
**PMI**						
Within 1 day	6	7	6	3	20	8
From 1 to 3 days	12	2	7	4	16	11
Over 3 days	5	2	8	2	4	7

**Figure 6:**
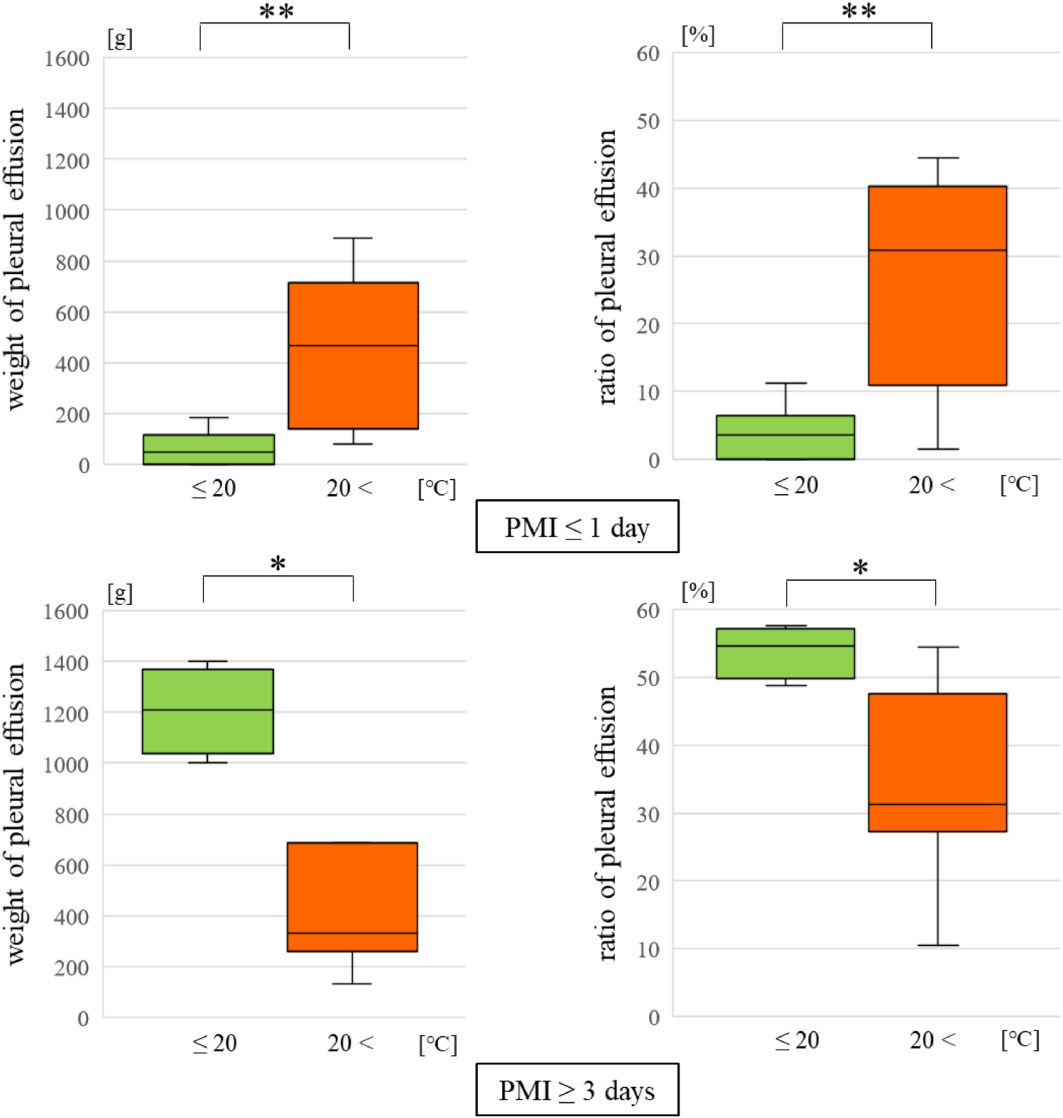
Comparing the weight and the ratio of pleural effusion for different water temperatures in the seawater group.

Cases were classified by both PMI and drowning environment to confirm the influence of water temperature. There were significant differences only in seawater. In cases with PMI ≤ 1 day, the PE weight and ratio increased in the 20< °C group (p < 0.01). In contrast, in cases with PMI ≥ 3 days, these parameters decreased in the 20< °C group (p < 0.05) (Fig. 6).

### Trauma, drugs, and ethanol

Each examined parameter was compared between the ethanol-positive and ethanol-negative groups. No significant differences were observed between the two groups in terms of trauma, drugs, or chemicals.

## Discussion

In this study, we investigated cases of drowning diagnosed by autopsy. Each case had aspirated a substantial amount of water, enough to cause death. The increase in intrathoracic weight depends on the volume of aspirated water, as water in the lungs leaks out to the pleural cavity due to postmortem changes. We examined several factors related to the volume of drowning water.

Seawater has a salinity of 3.5% and causes the withdrawal of water from the plasma into the lungs. Subsequently, lung oedema worsens, leading to fatality. In contrast, in freshwater, the transport of hypotonic liquid into the circulation causes hypervolemia, hemolysis with intravascular K^+^ release, and ventricular fibrillation^2^. Ultrastructural alterations of the lungs in cases of drowning in freshwater and seawater have been analysed in experimental drowning models^8–10^. The changes in freshwater drowning seemed to be predominantly osmotic in nature, as evidenced by severe cellular disruption, mitochondrial swelling, and endothelial destruction. These alterations indicate that the destruction of the lungs is more severe in freshwater than in seawater. Thus, the volume of drowning water may be attributable to the pathophysiological differences between seawater and freshwater drowning; less in freshwater, more in the higher salinity of the seawater.

The weight of the lungs was lightest, and the weight and the ratio of PE were heaviest in the ≥ 3 days group. In contrast, the IT weights showed no significant differences. These results confirm that drowning water in the lungs leaks out into the pleural cavity due to postmortem changes. Moreover, the leak was remarkable in the group with PMI ≥ 3 days.

According to the PMI, the parameters changed in drowning cases in seawater and freshwater, but not brackish water. This tendency was greater in seawater, because the weight of the lungs was heavier, representing a higher volume of water. Furthermore, osmotic pressure is also related to water leakage into the thoracic cavity. However, in brackish water, the PE weight varied irrespective of the postmortem intervals. This may be attributable to a wide salinity range between 0.5–1.5% with the ride and flow of the tides in brackish water.

We compared the weights and ratio based on average water temperature where each cadaver was first found (17.9 ± 7.6 ℃). The intrathoracic weight was lower in the < 18 °C group in brackish water (p < 0.05) (data not shown). The locations in which the cadavers were found included rivers, ponds, and irrigation ditches in the fresh and brackish water drownings environments. The water temperature of these places is influenced by many factors, such as air temperature, amount of precipitation, water discharge, and quantity of water intake. These factors may affect the cadaver’s postmortem movement. There was no significant difference in the ratio of PE attributed to these complex environmental factors.

Subsequently, we focused on the seawater temperature. The average water temperature is 20.4 ± 4.2 ℃ (12.9–28.4 ℃) in the Kii-channel facing the Tokushima and Wakayama prefectures^11^. Thus, we divided the cases into two groups based on a cut-off temperature of 20 °C. The PE ratio was higher in the > 20 °C group than in the ≤ 20 °C group in seawater drownings. The process of postmortem changes is greatly affected by environmental temperature. Generally, the corpses sink in the water because of the specific gravity and resurface due to postmortem putrefactive gases. We focused on the time to resurface as an indicator for postmortem change of the corpses in the water^12^. As a result, there was no difference in the days to resurface when the water temperature was over 10 °C. The Kii-channel is relatively warm, and the speed to resurface might be similar throughout the year. Therefore, it was thought that the ratio of PE was influenced the seawater temperature simply compared with the other areas.

Comparing the water temperature and PMI, the parameters changed as the PMI increased in the ≤ 20 °C group. These changes prove that water in the lungs leaks out to the pleural cavity due to postmortem changes. In the > 20 °C group, there was no significant difference. It was thought that postmortem changes had progressed rapidly because of the high water temperature.

In cases of PMI ≤ 1 day, the PE weight and ratio were increased in the 20< °C group and the PMI ≥ 3 days cases, they were decreased in the 20< °C group in the seawater drownings. During drowning, water may leak out to the pleural cavity in < 1 day and PE may be absorbed by the chest wall due to postmortem changes over ≥ 3 days. This significant difference was observed only in seawater because of a larger amount of PE had been stored in other areas.

Previous studies have been conducted on lung weight. Chen et al. reported that postmortem lung weight may indicate a longer survival time, inducing congestion and oedema^13^, but this correlation was not observed in all drowning cases. The survival time in our cases was not obvious, but most of them may have been acute deaths. Thus, it was not necessary to consider the influence of the survival time. Zhu et al. reported that sex and age were related to the weight of the lungs and PE in drownings^14^. No significant correlations were observed in this study. It was thought that the types of statistical tests used may have caused this difference. Furthermore, it has been reported that lung weight is correlated to subject height (of living patients)^15^. We also investigated drowning cases in Wakayama and found a significant correlation (correlation coefficient = 0.32, p < 0.01). For these reasons, measuring only the weight of the lungs and PE may not be sufficient to diagnose the cause of death.

In forensic practice, we may encounter drowning cases with severe trauma (spinal cord injury, multiple fractures) and drugs/poisons, including ethanol. We hypothesized that the process of death and the volume of aspirated water might be different in such cases. Unexpectedly, each examined parameter demonstrated no influence on the death process or the volume of aspirated water. Forensic pathologists should always consider other findings, such as the degree of trauma or drug and ethanol concentrations in the blood, even if the weight of the pleural cavity of the cadaver is sufficient to diagnose the cause of death as drowning.

PE is an important finding of drowning. In this study, the PE ratio was effective in diagnosing drowning more clearly. Moreover, water temperature, drowning environment, and PMI affect the weight of the PE. It is important to assess the PE ratio and to consider these factors when performing an autopsy of immersed bodies.

## Methods

### Autopsy cases

A total of 130 drowning cases were collected from forensic autopsy samples and investigated in this study. The diagnosis was based on macroscopic and microscopic findings, toxicological examinations, and diatom tests. Cases with open pleural cavities, pleural adhesions, and cases involving infants, were excluded. A summary of the investigated cases is presented in Table 5.

**Table 5:** Summary of the cases.

	Male	Female	p value	Total
Sex	88	42		n = 130
Age	17 to 93	26 to 93		17 to 93 years old
	66.1 ± 16.6	65.1 ± 18.2	0.75	65.8 ± 17.1

### Groups (Table 6)

1. Drowning environment
  The cases were classified into the following three groups based on the water environment where each cadaver was found: freshwater, brackish water, and seawater.
2. Water temperature
  The cases were classified into the following two groups based on the water temperature where each cadaver was found: under 20 °C and more than 20 °C.
3. PMI
  The cases were classified into three groups according to PMI: < 1 day, 1–3 days, and > 3 days.
4. Trauma
  The cases were classified into two groups according to the presence of cervical cord and/or thoracic fractures.
5. Drugs and chemicals in the blood
  The cases were classified into two groups according to the presence of drugs and chemicals in the blood. Toxicological screening of the blood in the right atrium and/or femoral vein was performed using gas chromatography mass spectrometry (GC–MS) on a QP-2010Ultra (Shimadzu, Kyoto, Japan) and prominence liquid chromatograph (Shimadzu UFLC system, Kyoto, Japan) coupled to a TSQ Quantum Access MAX tandem mass spectrometer (LC–MS/MS) (Thermo Scientific, Waltham, MA, USA), according to our previous reports^16,17^.
6. Ethanol in the blood
  The cases were classified into two groups according to the blood ethanol level (less than 0.01 mg/ml). Ethanol analysis was performed by headspace gas chromatography with flame ionisation detection (HS-GC-FID) on a QP-2010Plus GC (Shimadzu, Kyoto, Japan)^18^.

**Table 6:** Case details classified to groups.

	Group 1	Group 2	Group 3
Drowning environment	Fresh	Brackish	Sea
	34	30	66
Water temperature	Under 20 °C	over 20 °C	
	84	46	
Postmortem interval	Within 1 day	From 1 to 3 days	Over 3 days
	50	52	28
Trauma	With trauma	Without trauma	
	11	119	
Drugs	Negative	Positive	
	103	27	
Ethanol	Negative	Positive	
	94	36	

### Examined parameters

We investigated the following parameters: (1) the weight of the lungs, (2) the weight of PE, (3) intrathoracic weight (total weight of the lungs + PE), and (4) the ratio of PE (the weight of PE/intrathoracic weight × 100).

### Statistical analysis

Statistical analysis was performed using Statcel-the Useful Addin Forms on Excel-4th ed. (OMS Publishing, Tokyo, Japan). The Tukey–Kramer test was used to evaluate significant differences.

### Ethical approval

Our study including protocols was approved by the Human Ethics Review Committee of Wakayama Medical University, and was carried out in accordance with the Declaration of Helsinki Principles. Moreover, this study was conducted using autopsy records from the past, and we could not obtain informed consent from the bereaved family for the use of the records. Therefore, in accordance with the "Ethical Guidelines for Medical Research Involving Human Subjects (enacted by the Ministry of Health, Labor and Welfare in Japan)," Section 12-1 (2) (a) (c).

### Study approval

All methods were performed in accordance with relevant guidelines and regulations.

## Data Availability

The authors declare that all data are available in the article file. All data are also available from the authors upon reasonable request.
